# May Ingestion of Leachate from Decomposed Corpses Cause Appendicitis? A Case Report

**DOI:** 10.1155/2011/467137

**Published:** 2011-03-16

**Authors:** Maurício Domingues-Ferreira, Pedro Saddi-Rosa, André Luis dos Santos

**Affiliations:** ^1^Hospital da Policia Militar, 04021-001 São Paulo, SP, Brazil; ^2^Universidade Federal de São Paulo, 04021-001 São Paulo, SP, Brazil; ^3^Universidade de Santo Amaro-UNISA, 2141-8500 São Paulo, SP, Brazil

## Abstract

The general consensus is that appendicitis is basically provoked by fecaliths or lymphoid hyperplasic obstruction. Several studies based on histological diagnosis have not confirmed this hypothesis. On the contrary, obstruction has been proved in only a minority of cases. Diverse infections by parasites, bacteria, fungus, and noninfective agents have been associated with appendicitis in the medical literature. We describe a firefighter, who ingested a small quantity of leachate from decomposing corpses while working and developed enteritis a few hours later, which lasted several days and evolved to appendicitis. This case raises the possibility that the high quantity of bacteria concentration present in the leachate could have provoked enteritis and the subsequent appendicitis due to a direct effect of the bacteria on the appendix.

## 1. Introduction

Common sense leads us to believe that appendicitis is mainly provoked by obstruction of the appendix lumen due to fecaliths or lymphoid hyperplasia, followed by secondary bacterial invasion of the wall. Numerous studies and reports based on histologic diagnosis have not confirmed this hypothesis, and obstruction of the appendix lumen as cause of appendicitis has been proven in only a minority of cases [1]. In fact, the causes of appendicitis are multiple and not fully elucidated [2]. Aside from the obstruction theory, it is believed that appendicitis can be provoked by ischemia of the extramural appendiceal vascular supply, mucosal ulceration due to viral infection, leading to bacterial superinfection, retention of stool in the appendix due to diminished intestinal transit provoked by low fiber intake, trauma, genetic susceptibility, foreign bodies, hygiene, ischemia, and Type 1 hypersensitivity [1, 2]. In this report, we describe a very remarkable case of appendicitis that is strongly related to accidental ingestion of leachate from a decomposed corpse. Leachate is a liquid produced by the action of water precipitation on waste deposited in a landfill. It can be produced by decomposed corpses, and it has high concentrations of bacterial components. This case report leads us to a provocative question: whether the ingestion of a high concentration of bacteria could cause appendicitis. 

## 2. Case Report

The patient was a 27-year-old, athletic, Caucasian male, with no previous health problems and a firefighter in the city of Guarulhos, within the metropolitan area of São Paulo, Brazil. In March 2004, his mission was to explore a 20 m deep clandestine cesspool that was used to hide corpses of the victims of a local criminal gang. There were at least five advanced decomposed corpses at the bottom of the cesspool. He descended into the cesspool to retrieve the corpses, wearing special clothing and a mask to protect him from biological contamination from the decomposing corpuses. He spent about 30 minutes working inside the cesspool. Strong rain occurred while performing the task and his mask and his special cloths were damaged, and his body and his mask were covered in contaminated leachate from the decomposing corpuses. This situation led him accidentally swallow a small quantity of this leachate mixed with rain water while he was doing his job. Upon completion of the mission, he was dispensed from duty. About four hours later, he started to feel very sick and presented vomiting, diffuse abdominal pain, important diarrhea, and prostration. After 36 hours, the vomiting and the diarrhea had ceased, but abdominal distension manifested in association with constipation, nausea, loss of appetite, and diffuse abdominal pain. Sometimes, the level of abdominal pain increased to the point where he was obliged to seek medical assistance at the emergency unit for administration of analgesics and antiemetics. He never presented fever. This scenario lasted about 13 days when the diarrhea returned associated with fecaloid vomiting. Two days later the diarrhea ceased, the constipation restarted, flatus ceased, and the diffuse abdominal pain evolved into mesogastric pain. The vomiting became uncontrollable. He was hospitalized and submitted to an exploratory laparotomy, during which he was diagnosed with retrocecal appendicitis associated with intra-abdominal abscess and adhesions and was submitted to surgery to remove the same. The anatomopathological exam verified suppurative acute appendicitis and periappendicitis, with no evidence of fecaliths. Microscopic examination revealed neutrophilic infiltration of the mucosa, submucosa, and muscularis associated with areas of ulceration of the mucosa and abscess in the appendix wall presenting necrosis. He was drained and treated with antibiotics (amikacin, metronidazole, and cephalothin). After surgery, he began to feel progressive abdominal pain again, distension, constipation, and no fever. These symptoms got worse, and after 14 days he underwent a second surgery during which an intra-abdominal abscess and peritonitis were observed. One month later, he presented intestinal obstruction and abdominal pain and was submitted to a third operation due to another intra-abdominal abscess and adhesion. One year later, he was again submitted to surgery due to intestinal occlusion and incisional hernia. Six months after that, he underwent a fifth surgery for abdominal adhesion and intestinal occlusion. After the first surgery, he began to suffer chronic constipation that worsened after each successive surgery. Nowadays, he is only able to defecate after enteroclysis, to which he must be submitted every five days. 

## 3. Discussion

What is most remarkable in this case is the fact that only a few hours after the patient had ingested a contaminated leachate solution, an illness suggestive of bacterial gastroenteritis initiated that lasted 13 days and evolved to appendicitis. This could be considered a coincidence, but the fact that a coherent nexus exists linking the moment of contamination, the gastroenteritis, and the appendicitis is undeniable. Another interesting aspect is no fecaliths were detected in the appendix, and there is no proof that his appendicitis was provoked by fecalith obstruction. This report leads us to an intriguing possibility that ingestion of a high concentration of bacteria could provoke appendicitis. Specific bacterial infection may cause appendicitis, with or without involvement of the surrounding bowel [2]. The medical literature contains numerous reports associating intestinal infection by *Salmonella *(*typhoid* and *nontyphoidal*), *Yersinia* [2], *Bacteriodes* [3, 4], *Campylobacter* [5, 6], *Shigella *[7], and *Clostridium difficile* with appendicitis [8, 9]. Some authors believe that *Salmonella* infection could cause appendicitis by direct invasion of the appendix without the initial obstructive component. This bacterium colonizes the appendix mucosa and submucosa, provoking abscess and phlegmon [10]. *Yersinia* characteristically causes granulomatous appendicitis, which may or not be associated with enterocolitis and mesenteric adenitis [2]. Acute inflammation of the appendix provoked by *Campylobacter jejuni* is limited to the mucosa and submucosa, lacking transmural suppuration. Campbell evaluated 50 archival cases of acute appendicitis using molecular detection of *Campylobacter jejuni* and detected *Campylobacter jejuni* DNA in a significant percentage (22%) of acute appendicitis cases [11]. Appendiceal involvement in *Clostridium difficile *infection is very rare, and the pathological findings are similar to pseudomembranous colitis [2, 8, 9]. *Actinomyces* is a gram-positive, anaerobic, gut commensal that causes infection and predominantly neutrophilic inflammation of the appendix [2]. The most predominant bacteria detected in decomposed corpses and recent postmortems are *Clostridium perfringens*, enterobacteriaceae, streptococci, micrococcaceae, staphylococci, *Corynebacterium*, *Bacterioides*, *Pseudomonas*, *Alcaligenes*,* Achromobacter*, and occasionally bacilli [12]. Many of the bacteria related to appendicitis are present in decomposed corpses and could have been ingested in high concentrations by the patient. These ingested bacteria could be responsible by his gastroenteritis and could have triggered his appendicitis.

Another possible explanation for the patient's appendicitis is related to enteric lymphoid proliferation. Intestinal mucus is a fundamental and complex immunological organ. The entire intestine and appendix mucus presents an important lymphoid tissue that constantly reacts to antigens on the intestinal lumen. Some researchers believe the appendix has an important immunological role [13]. Bacteria express many different surface antigens and secrete a variety of virulence factors (bacterial endotoxins and exotoxins) that are highly immunogenic, and the ingestion of a high concentration of bacteria has the potential to trigger an intense immune response, which may be characterized by a huge proliferation of appendix lymphoid tissue. This lymphoid proliferation could be sufficient to produce an obstruction of the appendix lumen with subsequent bacterial proliferation, diminished venous and lymphatic drainage, ischemia, and appendicitis. Obstruction due to lymphoid proliferation has also been suggested as a possible explanation for appendicitis provoked by virus [14]. Lamps mentioned that appendicitis caused by parasites could be provoked by obstruction of the appendix lumen due to lymphoid hyperplasia that occurs in response to parasite antigens [2].

Moreover, Yildiz and Bulut reported a possible lymphoid hyperplasia appendicitis triggered by a barium enema performed 15 days prior to its manifestation [15]. 

After the surgery, the recurrence of intra-abdominal abscesses and adherences may reflect the high virulence intensity of the infection and the inflammatory process that the patient suffered. The sequelae developed due to multiple surgeries, included an important chronic constipation, probably the result of adherences, and dysfunction of intestinal peristalsis.

Another interesting aspect of this case is the fact that the patient's appendicitis was provoked by accidental ingestion of a substance with a high bacterial concentration while working; a situation that could be characterized as an occupational disease. We believe that this could be the first case report of appendicitis related to an occupational cause. 
